# Long non-coding RNA TUG1 promotes endometrial cancer development via inhibiting miR-299 and miR-34a-5p

**DOI:** 10.18632/oncotarget.15607

**Published:** 2017-02-22

**Authors:** Lifen Liu, Xin Chen, Ying Zhang, Yanrong Hu, Xiaoqing Shen, Weipei Zhu

**Affiliations:** ^1^ Department of Gynecology and Obstetrics, The Second Affiliated Hospital of Soochow University, Suzhou 215004, Jiangsu, China

**Keywords:** lincRNA, endometrial cancer

## Abstract

It is generally known that the human genome makes a large amount of noncoding RNAs compared with coding genes. Long non-coding RNAs (lncRNAs) which composed of more than 200 nucleotides have been described as the largest subclass of the non-coding transcriptome in human noncoding RNAs. Existing research shows that lncRNAs exerted biological functions in various tumors via participating in both oncogenic and tumor suppressing pathways. The previous studies indicated that lncRNA taurine upregulated 1 (TUG1) play important roles in the initiation and progression of malignancies. In this study,based on previous research, we investigated the expression and biological role of the lncRNA-TUG1. We analyzed the relationship between lncRNA-TUG1and endometrial carcinoma (EC) in a total 104 EC carcinoma specimens, compared with that in normal tissues. We found that lncRNA-TUG1 expression in cancer tissues was significantly higher than that in adjacent tissues. Through a series of experiments, the results demonstrated that lncRNA-TUG1 enhances the evolution and progression of EC through inhibiting miR-299 and miR-34a-5p.

## INTRODUCTION

Endometrial carcinoma (EC), the most common form of gynecological malignancy, which can be divided into two types: type I, estrogen-dependent endometriosis carcinoma; and type II, estrogen-independent non-endometriosis carcinoma [1–3]. The type I usually have favorable prognosis while type II endometrial cancers are more aggressive and also presented poor prognosis [4]. There have been many comparative studies demonstrate that epigenetic changes closely associated with the occurrence and development of EC, finding the new biomarkers for metastatic progression in EC is urgent.

Long non-coding RNAs (lncRNAs) which composed of more than 200 nucleotides, usually do not have the capacity to encoding protein [5, 6]. Formerly, some lncRNAs have been discovered in what had been considered junk but a rapidly growing number of recent studies show that lncRNAs as new modulators in the tumorigenesis and progression in cancer by participating in both oncogenic and tumor suppressing pathways [7, 8]. Recently, lncRNAs as a competing endogenous RNA (ceRNA) and sponge miRNAs, regulating the expression of target mRNA is becoming a new hotspot of epigenetics research [9].

Taurine upregulated 1 (TUG1), a novel lncRNA with 6.7-kb nucleotides, which was initially characterized by a genomic screening study in mouse retinal cells [10]. LncRNA TUG1 was frequently upregulated and characterized as an oncogene involved in the development and progression of a variety of tumors (e.g., osteosarcoma [11], esophageal squamous cell carcinoma (ESCC) [12], urothelial carcinoma of the bladder [13], glioblastoma [14], colorectal cancer [15]). Previous studies indicated that lncRNA TUG1 through regulate the expression of the miRNA and its target genes involved in the development and progression of cancer. However, the biological functions of lncRNA TUG1 in the control of EC tumorigenesis have not been well characterized, which prompted us to explore the role of lncRNA TUG1 in human EC. In this study, we delineate the transcriptional aberration of lncRNA TUG1 in EC tumor tissues and corresponding adjacent normal tissues

## RESULTS

### Cellular location of lncRNA-TUG1

**Figure 1:**
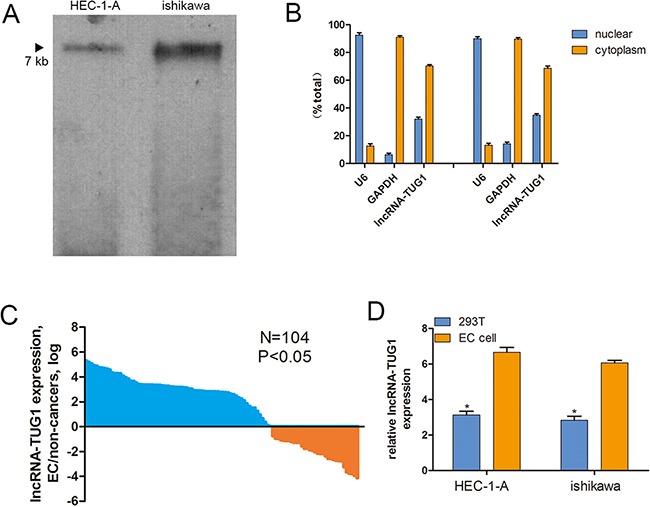
Cellular and molecular characterization of lncRNA-TUG1 **A**. Northern blot analysis of lncRNA-TUG1 expression in EC cells. **B**. The levels of nuclear control transcript (U6), cytoplasmic control transcript (GAPDH mRNA) and lncRNA-TUG1were assessed by qRT-PCR in nuclear and cytoplasmic fractions. Data are mean ± SEM. **C**. The lncRNA-TUG1 expression was higher in EC tumor tissues than the adjacent tissues, the expression level of *lincRNA-uc002kmd.1* was analyzed by qRT-PCR normalized to *GAPDH*. Data are represented as mean ± SEM. **D**. LncRNA-TUG1 endogenous expression levels in EC cell lines were determined by quantitative real-time PCR normalized to 293T using GAPDH as an internal control.

Firstly, we conducted northern blot experiments to detect the transcript of lncRNA-TUG1, the result showed that TUG1 is a 6.7-kb transcript in EC cells (Figure 1A). Next, we address the cellular localization of lncRNA-TUG1, the levels of the nuclear control transcript (*U6*) and cytoplasmic control transcript (*GAPDH* mRNA) were detected by RT-qPCR in the nuclear and cytoplasmic fractions, respectively. The results showed that lncRNA-TUG1 mostly distributed in cytoplasm of EC cells. For the HEC-1-A cell line, qRT-PCR analysis revealed that (mean ± SEM) 70.3% lncRNA-TUG1 was detected in the cytoplasmic fraction, and 32.1% was situated in the nuclear fraction. Similar results were obtained with the ishikawa cell line, 68.6% lncRNA-TUG1 was detected in the cytoplasmic fraction, and 34.8% was situated in the nuclear fraction (Figure 1B).

### Expression of lncRNA-TUG1 in EC tissues

We detected lncRNA-TUG1 expression in 104 paired EC tissue and corresponding adjacent normal tissue. The results showed that the lncRNA-TUG1 expression was higher in 71 tumor tissues (68.27%) than the adjacent tissues (*P* < 0.05; Figure 1C), and we find that there is no significance differential expression in subgroups of patients too (P>0.05, Supplementary Figure 1). In addition, the lncRNA-TUG1 expression level was significantly increased in endometrial carcinoma cells compared with normal human 293T cell (*P<0.05*; Figure 1D).

### Knockdown lncRNA-TUG1 in EC cell lines

**Figure 2:**
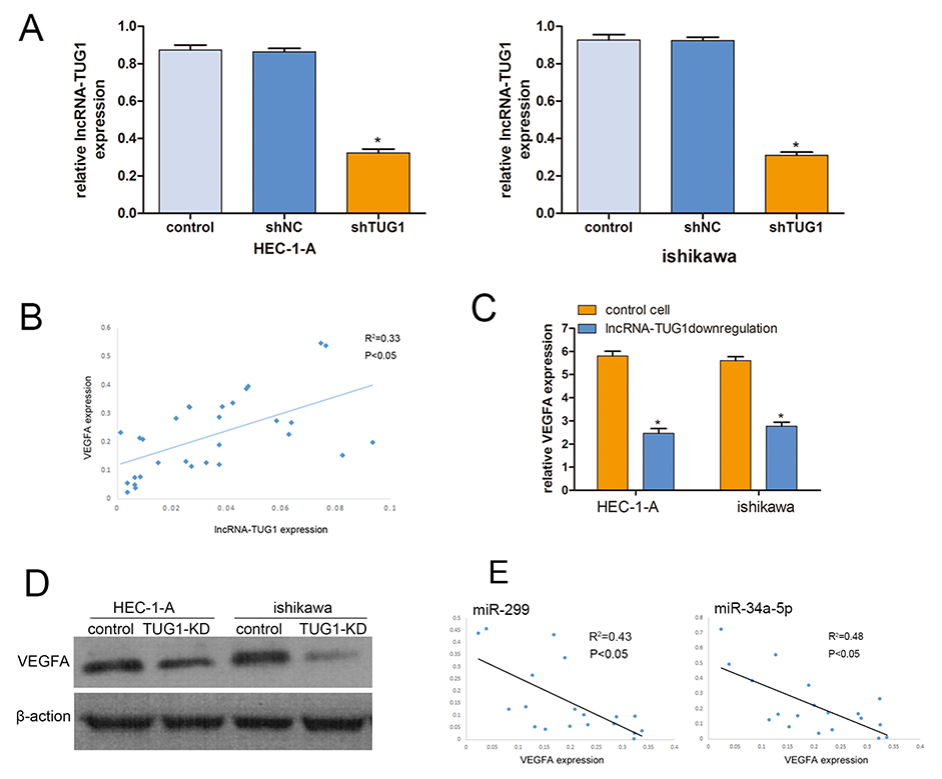
Knockdown lncRNA-TIG1 in EC cells **A**. HEC-1-A and ishikawa cells were transfected with lncRNA-TUG1 siRNA and control siRNA for 48 h. qRT-PCRs were conducted to detect lncRNA-TUG1 levels. The data were shown as fold increase compared with control group. Data are mean ± SEM, normalized to GAPDH. **B**. The linear correlations between the lncRNA-TUG1expression levels and VEGFA mRNA were tested. The relative expression value was normalized by GAPDH expression level. **C**. The expression of VEGFA was detected after downregulated lncRNA-TUG1. **D**. The protein levels of VEGFA was assessed in HEC-1-A cells and ishikawa cells by Western blot. **E**. The linear correlations between the miR-299 and miR-34a-5p expression levels and VEGFA mRNA were tested.

In order to study the role of lncRNA-TUG1 in EC cells, we construct the shRNA-TUG1 and silencing control plasmid (shNC). Quantitative real-time PCR analysis was applied to detect lncRNA-TUG1 expression and validate the efficiency of TUG1 knockdown. There was no significant difference in lncRNA TUG1 expression between the control and shNC groups. Reversely, lncRNA TUG1 expressed 62.8% lower in the shTUG1 group than that in shNC group of HEC-1-A cells, while it was 66.31% lower in the shTUG1 group than that in shNC group of ishikawa cells (*P<0.05*) (Figure 2A).

### Association of lncRNA-TUG1 and VEGFA in EC

To test the correlation between lncRNA-TUG1 and VEGFA, we choose another 30 endometrial carcinoma tissues. The results showed that patients with higher lncRNA-TUG1 expression levels in EC tissue displayed substantial up-regulation of VEGFA (*R^2^* = 0.33, *P* < 0.05; Figure 2B).

The expression of VEGFA decreased significantly in TUG1 knockdown cells compared with the normal control. (Figure 2C). Western Blot results consistently showed that knockdown of lncRNA-TUG1 decreased VEGFA protein levels in the HEC-1-A cell line. Similar results were found in the ishikawa cell line (Figure 2D).

### lncRNA-TUG1 regulates VEGFA expression by competing for miR-299 and miR-34a-5p

**Figure 3:**
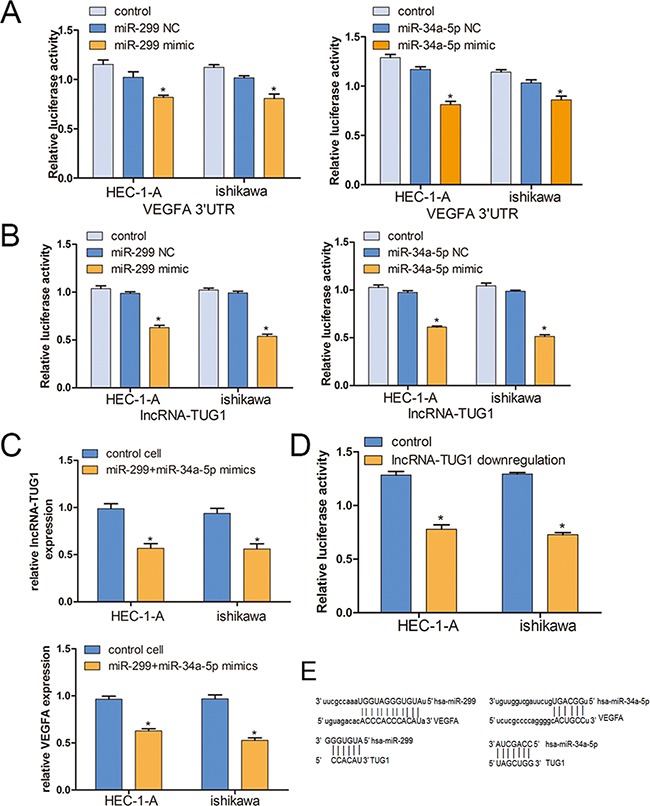
lncRNA-TUG1 and VEGFA are targeted by miR-299 and miR-34a-5p **A, B**. Both lncRNA-TUG1 and VEGFA are targeted by miR-299 and miR-34a-5p. Relative luciferase activity was performed by dual-luciferase reporter assay. Data represent mean ± SEM. (n =3, each). **C**. The expression levels of lncRNA-TUG1 and VEGFA in the EC cells after treating with the miRNA mimics. **D**. The lncRNA-TUG1 shRNA reporter vector and control vector were co-transfected to EC cells, the VEGFA luciferase signal was significantly decreased(P<0.05). **E**. Computational miRNA target prediction analysis.

In the previous study, lncRNA-TUG1 acted as a competing endogenous RNA (ceRNA) through modulating the expression and biological functions of miRNA [14, 16]. Coincidentally, the miR-299 and miR-34a-5p was the experimental verified target for both lncRNA-TUG1 and VEGFA [14, 17]. We test the correlation between miRNAs and VEGFA, we choose another 20 endometrial carcinoma tissues. The results showed that patients with higher miRNA expression levels in EC tissue displayed substantial down-regulation of VEGFA (*R^2^* = 0.43, *P* < 0.05 for miR-299; *R^2^* = 0.48, *P* < 0.05 for miR-34a-5p; Figure 2E). To test the role of miR-299 and miR-34a-5p in the EC cells, the 3′ UTR of VEGFA and TUG1 cDNA was cloned into the downstream of luciferase gene and transfected into EC cells with miR-299 and miR-34a-5p mimics respectably. The results showed that both miR-299 and miR-34a-5p significantly decreased the luciferase signals of the mentioned reporters above (Figure 3A, B). Moreover, qPCR assay was used to test the expression levels of lncRNA-TUG1 and VEGFA in the EC cells after treating with the miRNA mimics. Not surprisingly, both the lncRNA-TUG1 and VEGFA levels were significantly decreased (Figure 3C).

Next, we cloned the 3′UTR region of into a luciferase reporter and co-transfected in the lncRNA-TUG1 knockdown cells. The results showed that the fluorescent value of VEGFA in lncRNA-TUG1 knockdown cells is significantly lower than the control side (p<0.05). All above results suggested that lncRNA-TUG1 regulates VEGFA expression by competing for miR-299 and miR-34a-5p (Figure 3D). The predict potential binding sites of lncRNA-TUG1 and 3′UTR of VEGFA with miR-299 and miR-34a-5p were showed in Figure 3E.

### lncRNA-TUG1 modulates cell growth

**Figure 4:**
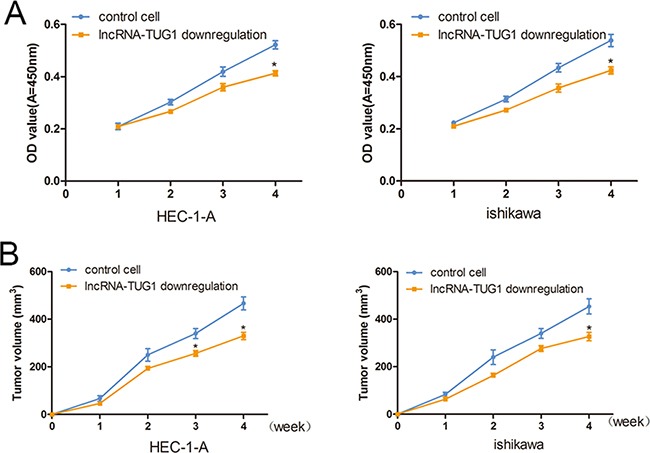
lncRNA-TUG1 mediated cell proliferation in EC cells **A**. HEC-1-A and ishikawa cells viability were measured by CCK-8 proliferation assay. Six replicates for each group and the experiment repeated three times. Data are mean±SEM. * *P*<0.05 compared with controls. **B**. Stable EC cells were injected subcutaneously into nude mice. Mean tumor volumes from six nude mice of each group are shown at different time points. * *P*<0.05 compared with controls.

The effects of lncRNA-TUG1 on the EC cells proliferation were checked by CCK-8 assays. there was no significant difference in the viability of EC cells in the control group. Reversely, the viability of EC cells was significantly lower in lncRNA-TUG1 down-regulated cells (HEC-1-A was 21% decrease and ishikawa was 21.3% decrease) (Figure 4A).

### LncRNA-TUG1 knockdown inhibits tumor growth

To examine the biological significance of lncRNA-TUG1 on tumor growth, xenograft was subcutaneously injected with EC cells. As is shown in Figure 4B, the growth of tumors from lncRNA-TUG1-downregulated xenografts were significantly inhibited compared with that of the control xenografts (466.7 ± 38.6 mm^3^ versus 330 ± 21.6 mm^3^ for HEC-1-A cells (*P* < 0.05); and 453.3 ± 44.9 mm^3^ versus326.7± 24.9 mm^3^ for ishikawa cells (*P* < 0.05)).

## DISCUSSION

In our study, we detected the lncRNA-TUG1 expression in EC, the results showed that it was dramatically up-regulated in EC tissues and then we further explored functional role and possible mechanism of lncRNA-TUG1 in EC. Through a series of experiments, we concluding that lncRNA-TUG1 act as one competitive endogenous

RNAs (ceRNA) negative regulate the miR-299 and miR-34a-5p and influence the expression of VEGFA in EC.

In recent years, with the development of whole-genome and transcriptome sequencing technologies, lncRNAs have been increasingly concerned and emphasized by researchers [8, 18]. To the best of our knowledge, lncRNAs are emerging as key regulators in cell biology, and mountains research have proposed that the link between their abnormal expression and diverse human diseases, especially in cancer [19–22]. For instance, the most commonly known lncRNA, HOTAIR, is up-regulated in gallbladder cancer (GBC) that leads to tumor metastases through altered methylation of histone H3 lysine 27 (H3K27) and gene expression [23, 24]. LncRNA-MEG3 is down-regulated in EC by repressing Notch signaling pathway [25]. Linc-POU3F3 is increased in ESCC tissues and contributes to development of cancer through interactions with EZH2 to promote methylation of POU3F3 [26]. In the present study, we focus on the lncRNA-TUG1, which is abnormal expressed in a wide variety of tumors, and we also conducted a series of experiments to explore the role of lncRNA-TUG1 acted in EC development. We conduct lncRNA-TUG1 knockdown assay, the results show that silence lncRNA-TUG1 inhibits tumor growth *in vivo*, and reduces EC cell proliferation *in vitro*.

Recently, a novel regulatory mechanism between lncRNAs and miRNAs has received attention by more and more researchers. LncRNAs may act as miRNA sponges participate in the competitive endogenous RNAs (ceRNA) regulatory network to negatively regulating the miRNA expression and then influence the expression of miRNA target gene [27, 28]. For instance, GAPLINC improve CD44 expression by competing for miR-211-3p, subsequently mediating cell migration and proliferation in gastric carcinoma [29]. LncRNA urothelial carcinoma-associated 1(lncRNA-UCA1) through inhibition of miR-216b and activation of FGFR1/ERK signaling pathway in hepatocellular carcinoma [30]. Identically, there was a reciprocal repression between lncRNA-TUG1 and miR-299, lncRNA-TUG1-miR-299 constitutes a ceRNA regulatory network in the progress of glioblastoma. Also, lncRNA-TUG1 act as miR-34a-5p sponge in hepatoblastoma [17]. Coincidentally, our data also affirmed that lncRNA-TUG1 forms a molecular decoy for miR299 and miR-34a-5p in EC cells.

MicroRNAs are members of the class of non-coding RNAs, which often emerged as regulators of gene expression. Aberrant expression of miR-299 was determined in various kind of cancers, such as breast cancer and metastatic prostate cancer [31]. Past researchers have found that overexpression of miR-299 could suppress the proliferation of metastatic prostatic cancer cells [32]. Similarly, miRNA-34a has been reported as tumor suppressor in multiple cancer types, like neuroblastoma [33], colon cancer [34], and prostate cancer [35]. In our study, we verified the roles of miR-299 and miRNA-34a in endometrial cancer progression, the results of dual-luciferase reporter assay show that both two miRNAs could direct regulate VEGFA expression in EC cells. At the same time, we also find a positive correlation between the expression of VEGFA and lncRNA-TUG1.

There is a great deal of research shows that vascular endothelial growth factor (VEGF) family, which comprising VEGF-A to –D and their receptors VEGFR1 (flt-1), VEGFR2 (KDR/flk-1) and VEGFR3 (flt-4) is the critical regulator in angiogenesis signaling in a variety of tumors [36, 37]. VEGFA as the member of VEGF family has been identified as the predominant tumor angiogenesis factor in the majority of human cancers. In our lncRNA-TUG1 knockdown cells, the expression level of VEGFA was significantly reduced in the presence of miR-299 and miR-34a-5p, suggesting that lncRNA-TUG1 may functions as a ceRNA to regulate VEGFA levels by sponging miRNA.

In conclusion, our present study firstly revealed that lncRNA-TUG1 was upregulated in EC tissues and cell lines. LncRNA-TUG1 may improve VEGFA expression by competing for miR-299 and miR-34a-5p, subsequently mediating cell proliferation in EC. The lncRNA-TUG1/miRNA/VEGFA network may become a candidate target for EC therapy.

## MATERIALS AND METHEDS

### Study subjects

Homogenous Han Chinese comprised the subjects participating in this study. To validate RNA expression data, we studied 104 paired EC tissues and matched non-cancerous tissues that were obtained during surgical resection from the affiliate hospitals of Soochow University (Suzhou, China). The diagnosis of EC was pathologically confirmed in all cases and the non-tumorous samples were taken at a distance of at least 5 cm from the tumor tissue. The Medical Ethics Committee of Soochow University approved this study. The clinical characteristics of all the patients were described in detail previously and listed in Table 1 [38].

**Table 1:** Clinicopathological characteristics of endometrial cancer patients in Chinese population

Characteristics	Suzhou population	
	N^a^	(%)
Age (years)		
≤65	53	(51.0)
>65	51	(49.0)
Age at menarche (years)		
≤11	7	(6.7)
11–16	67	(64.4)
≥16	30	(28.8)
Menopausal status		
Premenopausal	32	(30.8)
Postmenopausal	72	(69.2)
Family history of cancer		
Positive	8	(7.7)
Negative	96	(92.3)
BMI^b^		
≤25	41	(39.4)
>25	63	(60.6)
FIGO^c^ stage		
I	79	(76.0)
II	7	(6.7)
III	18	(17.3)
Histologic type		
Endometrioid	91	(87.5)
Non-endometrioid	13	(12.5)
Grade		
G1	44	(42.3)
G2	53	(51.0)
G3	7	(6.7)

^a^*N*, number of patients.

^b^BMI, body mass index.

^c^FIGO, The International Federation of Gynecology and Obstetrics.

### Cell culture

Human endometrial cancer cell lines, HEC-1-A and ishikawa were purchased from the Cell Bank of Type Culture Collection of the Chinese Academy of Sciences, Shanghai Institute of Cell Biology, and were passaged for less than 6 months after resuscitation. The cell culture procedures reference literature that has been published before [39]. Cells were cultured at 37°C in 5% CO_2_ in RPMI-1640 medium supplemented with 10% fetal bovine serum, penicillin and streptomycin in a 10-ml culture dish.

### RNA extraction and real-time quantitative polymerase chain reaction

The total RNA from the cells and tissues were isolated by using TRIzol® reagent (Invitrogen), according to the manufacturer's instructions. The relative gene expression of lncRNA TUG1 was determined using the ABI Prism 7500 sequence detection system (Applied Biosystems, Foster City, CA, USA). *GAPDH* was used as an internal standard control, and all the reactions were performed in triplicate [40, 41]. The primers used for qPCR amplification were as follows: lncRNA-TUG1 forward, 5′-CTGAAGAAAGGCAACATC-3′ and reverse, 5′-GTAGGCTACTACAGGATTTG-3′; GAPDH forward, 5′-AGCCACATCGCTCAGACAC-3′ and reverse, 5′-GCCCAATACGACCAAATCC-3′ [15].

### Construction of reporter plasmids

The method for the construction of reporter plasmids has been published before [29]. The pGL3 promoter vector (GENECHEM) was used to construct the plasmids pGL3-lncRNA-TUG1 and pGL3- VEGFA-3′UTR. All the constructs were verified by DNA sequencing.

### Transient transfections and luciferase assays

HEC-1-A and ishikawa cells were transfected with the reporter plasmids using Lipofectamine 2000 (Invitrogen, CA, USA), according to the manufacturer's instructions. Dual-Luciferase Reporter assay system (Promega, Madison, WI, USA) were used to measure the luciferase activity [42]. Three independent experiments were conducted and each group included 6 replicates.

### Western blot

Western blot analysis was conducted to assess VEGFA and β-action expression, the experiments were performed as described by as previously described [39]. The Western blotting analysis was repeated at least three times.

### Cell viability assay

The Cell Counting Kit-8 (CCK-8) system (Dojindo Laboratory, Kumamoto, Japan) was used to measure cell viability, according to the manufacturer's instructions [42]. There were 6 replicates for each group, and the experiments were repeated at least 3 times.

### Statistical analyses

The correlation between the expression of lncRNA-TUG-1 and the VEGFA gene in EC tissue was assessed using one-way analysis of variance and linear regression models. A probability value P<0.05was considered statistically significant. Differences between the groups were assessed using paired, 2-tailed Student's t-test. Data were expressed as means ± SEM, from at least three independent experiments.

## SUPPLEMENTARY MATERIALS FIGURE



## References

[1] Hakim SA, Raboh NM (2015). Immunohistochemical expression of glypican 3 in endometrial carcinoma and correlation with prognostic parameters. International journal of clinical and experimental pathology.

[2] Macwhinnie N, Monaghan H (2004). The use of P53, PTEN, and C-erbB-2 to differentiate uterine serous papillary carcinoma from endometrioid endometrial carcinoma. International journal of gynecological cancer.

[3] Matias-Guiu X, Catasus L, Bussaglia E, Lagarda H, Garcia A, Pons C, Munoz J, Arguelles R, Machin P, Prat J (2001). Molecular pathology of endometrial hyperplasia and carcinoma. Human pathology.

[4] Goff BA (2005). Uterine papillary serous carcinoma: what have we learned over the past quarter century?. Gynecologic oncology.

[5] Feng YZ, Shiozawa T, Miyamoto T, Kashima H, Kurai M, Suzuki A, Konishi I (2005). BRAF mutation in endometrial carcinoma and hyperplasia: correlation with KRAS and p53 mutations and mismatch repair protein expression. Clinical cancer research.

[6] Maher CA, Kumar-Sinha C, Cao X, Kalyana-Sundaram S, Han B, Jing X, Sam L, Barrette T, Palanisamy N, Chinnaiyan AM (2009). Transcriptome sequencing to detect gene fusions in cancer. Nature.

[7] Guerra E, Trerotola M, Dell' Arciprete R, Bonasera V, Palombo B, El-Sewedy T, Ciccimarra T, Crescenzi C, Lorenzini F, Rossi C, Vacca G, Lattanzio R, Piantelli M (2008). A bicistronic CYCLIN D1-TROP2 mRNA chimera demonstrates a novel oncogenic mechanism in human cancer. Cancer research.

[8] Wang KC, Chang HY (2011). Molecular mechanisms of long noncoding RNAs. Molecular cell.

[9] Sun C, Li S, Zhang F, Xi Y, Wang L, Bi Y, Li D (2016). Long non-coding RNA NEAT1 promotes non-small cell lung cancer progression through regulation of miR-377-3p-E2F3 pathway. Oncotarget.

[10] Young TL, Matsuda T, Cepko CL (2005). The noncoding RNA taurine upregulated gene 1 is required for differentiation of the murine retina. Current biology.

[11] Zhang Q, Geng PL, Yin P, Wang XL, Jia JP, Yao J (2013). Down-regulation of long non-coding RNA TUG1 inhibits osteosarcoma cell proliferation and promotes apoptosis. Asian Pacific journal of cancer prevention.

[12] Xu Y, Wang J, Qiu M, Xu L, Li M, Jiang F, Yin R, Xu L (2015). Upregulation of the long noncoding RNA TUG1 promotes proliferation and migration of esophageal squamous cell carcinoma. Tumour biology.

[13] Han Y, Liu Y, Gui Y, Cai Z (2013). Long intergenic non-coding RNA TUG1 is overexpressed in urothelial carcinoma of the bladder. Journal of surgical oncology.

[14] Cai H, Liu X, Zheng J, Xue Y, Ma J, Li Z, Xi Z, Li Z, Bao M, Liu Y (2016). Long non-coding RNA taurine upregulated 1 enhances tumor-induced angiogenesis through inhibiting microRNA-299 in human glioblastoma. Oncogene.

[15] Wang L, Zhao Z, Feng W, Ye Z, Dai W, Zhang C, Peng J, Wu K (2016). Long non-coding RNA TUG1 promotes colorectal cancer metastasis via EMT pathway. Oncotarget.

[16] Braconi C, Kogure T, Valeri N, Huang N, Nuovo G, Costinean S, Negrini M, Miotto E, Croce CM, Patel T (2011). microRNA-29 can regulate expression of the long non-coding RNA gene MEG3 in hepatocellular cancer. Oncogene.

[17] Dong R, Liu GB, Liu BH, Chen G, Li K, Zheng S, Dong KR (2016). Targeting long non-coding RNA-TUG1 inhibits tumor growth and angiogenesis in hepatoblastoma. Cell death & disease.

[18] Hung T, Chang HY (2010). Long noncoding RNA in genome regulation: prospects and mechanisms. RNA biology.

[19] Yang F, Huo XS, Yuan SX, Zhang L, Zhou WP, Wang F, Sun SH (2013). Repression of the long noncoding RNA-LET by histone deacetylase 3 contributes to hypoxia-mediated metastasis. Molecular cell.

[20] Orom UA, Derrien T, Beringer M, Gumireddy K, Gardini A, Bussotti G, Lai F, Zytnicki M, Notredame C, Huang Q, Guigo R, Shiekhattar R (2010). Long noncoding RNAs with enhancer-like function in human cells. Cell.

[21] Geisler S, Lojek L, Khalil AM, Baker KE, Coller J (2012). Decapping of long noncoding RNAs regulates inducible genes. Molecular cell.

[22] Shi X, Sun M, Liu H, Yao Y, Song Y (2013). Long non-coding RNAs: a new frontier in the study of human diseases. Cancer letters.

[23] Ma MZ, Li CX, Zhang Y, Weng MZ, Zhang MD, Qin YY, Gong W, Quan ZW (2014). Long non-coding RNA HOTAIR, a c-Myc activated driver of malignancy, negatively regulates miRNA-130a in gallbladder cancer. Molecular cancer.

[24] Steele G, Busse P, Huberman MS, LeClair JM, Falchuk ZM, Mayer RJ, Bothe A, Ravikumar TS, Stone M, Jessup JM (1991). A pilot study of sphincter-sparing management of adenocarcinoma of the rectum. Archives of surgery.

[25] Guo Q, Qian Z, Yan D, Li L, Huang L (2016). LncRNA-MEG3 inhibits cell proliferation of endometrial carcinoma by repressing Notch signaling. Biomedicine & pharmacotherapy = Biomedecine & pharmacotherapie.

[26] Li W, Zheng J, Deng J, You Y, Wu H, Li N, Lu J, Zhou Y (2014). Increased levels of the long intergenic non-protein coding RNA POU3F3 promote DNA methylation in esophageal squamous cell carcinoma cells. Gastroenterology.

[27] Wang J, Liu X, Wu H, Ni P, Gu Z, Qiao Y, Chen N, Sun F, Fan Q (2010). CREB up-regulates long non-coding RNA, HULC expression through interaction with microRNA-372 in liver cancer. Nucleic acids research.

[28] Wang P, Liu YH, Yao YL, Li Z, Li ZQ, Ma J, Xue YX (2015). Long non-coding RNA CASC2 suppresses malignancy in human gliomas by miR-21. Cellular signalling.

[29] (2015). Correction: Long Noncoding RNA GAPLINC Regulates CD44-Dependent Cell Invasiveness and Associates with Poor Prognosis of Gastric Cancer. Cancer research.

[30] Wang F, Ying HQ, He BS, Pan YQ, Deng QW, Sun HL, Chen J, Liu X, Wang SK (2015). Upregulated lncRNA-UCA1 contributes to progression of hepatocellular carcinoma through inhibition of miR-216b and activation of FGFR1/ERK signaling pathway. Oncotarget.

[31] Azarbarzin S, Hosseinpour Feizi MA, Safaralizadeh R, Ravanbakhsh R, Kazemzadeh M, Fateh A, Karimi N, Moaddab Y (2016). The Value of miR-299-5p in Diagnosis and Prognosis of Intestinal-Type Gastric Adenocarcinoma. Biochemical genetics.

[32] Formosa A, Markert EK, Lena AM, Italiano D, Finazzi-Agro E, Levine AJ, Bernardini S, Garabadgiu AV, Melino G, Candi E (2014). MicroRNAs, miR-154, miR-299-5p, miR-376a, miR-376c, miR-377, miR-381, miR-487b, miR-485-3p, miR-495 and miR-654-3p, mapped to the 14q32.31 locus, regulate proliferation, apoptosis, migration and invasion in metastatic prostate cancer cells. Oncogene.

[33] Cole KA, Attiyeh EF, Mosse YP, Laquaglia MJ, Diskin SJ, Brodeur GM, Maris JM (2008). A functional screen identifies miR-34a as a candidate neuroblastoma tumor suppressor gene. Molecular cancer research.

[34] Siemens H, Neumann J, Jackstadt R, Mansmann U, Horst D, Kirchner T, Hermeking H (2013). Detection of miR-34a promoter methylation in combination with elevated expression of c-Met and beta-catenin predicts distant metastasis of colon cancer. Clinical cancer research.

[35] Lodygin D, Tarasov V, Epanchintsev A, Berking C, Knyazeva T, Korner H, Knyazev P, Diebold J, Hermeking H (2008). Inactivation of miR-34a by aberrant CpG methylation in multiple types of cancer. Cell cycle.

[36] Kieran MW, Kalluri R, Cho YJ (2012). The VEGF pathway in cancer and disease: responses, resistance, and the path forward. Cold Spring Harbor perspectives in medicine.

[37] Wang J, Taylor A, Showeil R, Trivedi P, Horimoto Y, Bagwan I, Ewington L, Lam EW, El-Bahrawy MA (2014). Expression profiling and significance of VEGF-A, VEGFR2, VEGFR3 and related proteins in endometrial carcinoma. Cytokine.

[38] Li N, Zheng J, Li H, Deng J, Hu M, Wu H, Li W, Li F, Lan X, Lu J, Zhou Y (2014). Identification of chimeric TSNAX-DISC1 resulting from intergenic splicing in endometrial carcinoma through high-throughput RNA sequencing. Carcinogenesis.

[39] Huang G, Zhu H, Shi Y, Wu W, Cai H, Chen X (2015). cir-ITCH plays an inhibitory role in colorectal cancer by regulating the Wnt/beta-catenin pathway. PloS one.

[40] Lehmann U, Kreipe H (2001). Real-time PCR analysis of DNA and RNA extracted from formalin-fixed and paraffin-embedded biopsies. Methods.

[41] Wu H, Deng J, Zheng J, You Y, Li N, Li W, Wu D, Zhou Y (2015). Functional polymorphisms in the CD44 gene and acute myeloid leukemia cancer risk in a Chinese population. Molecular carcinogenesis.

[42] Li N, Zhou P, Zheng J, Deng J, Wu H, Li W, Li F, Li H, Lu J, Zhou Y, Zhang C (2014). A polymorphism rs12325489C>T in the lincRNA-ENST00000515084 exon was found to modulate breast cancer risk via GWAS-based association analyses. PloS one.

