# Supplementary material for: Long non-coding RNA TUG1 promotes endometrial cancer development via inhibiting miR-299 and miR-34a-5p
Source: Oncotarget. 2017 Feb 22;8(19):31386–94. doi: 10.18632/oncotarget.15607 (PMC5458215; doi:10.18632/oncotarget.15607)
Supplement: Supplementary file 1 [file oncotarget-08-31386-s001.pdf]

Long non-coding RNA TUG1 promotes endometrial cancer development via inhibiting miR-299 and miR-34a-5p

SUPPLEMENTARY FIGURE

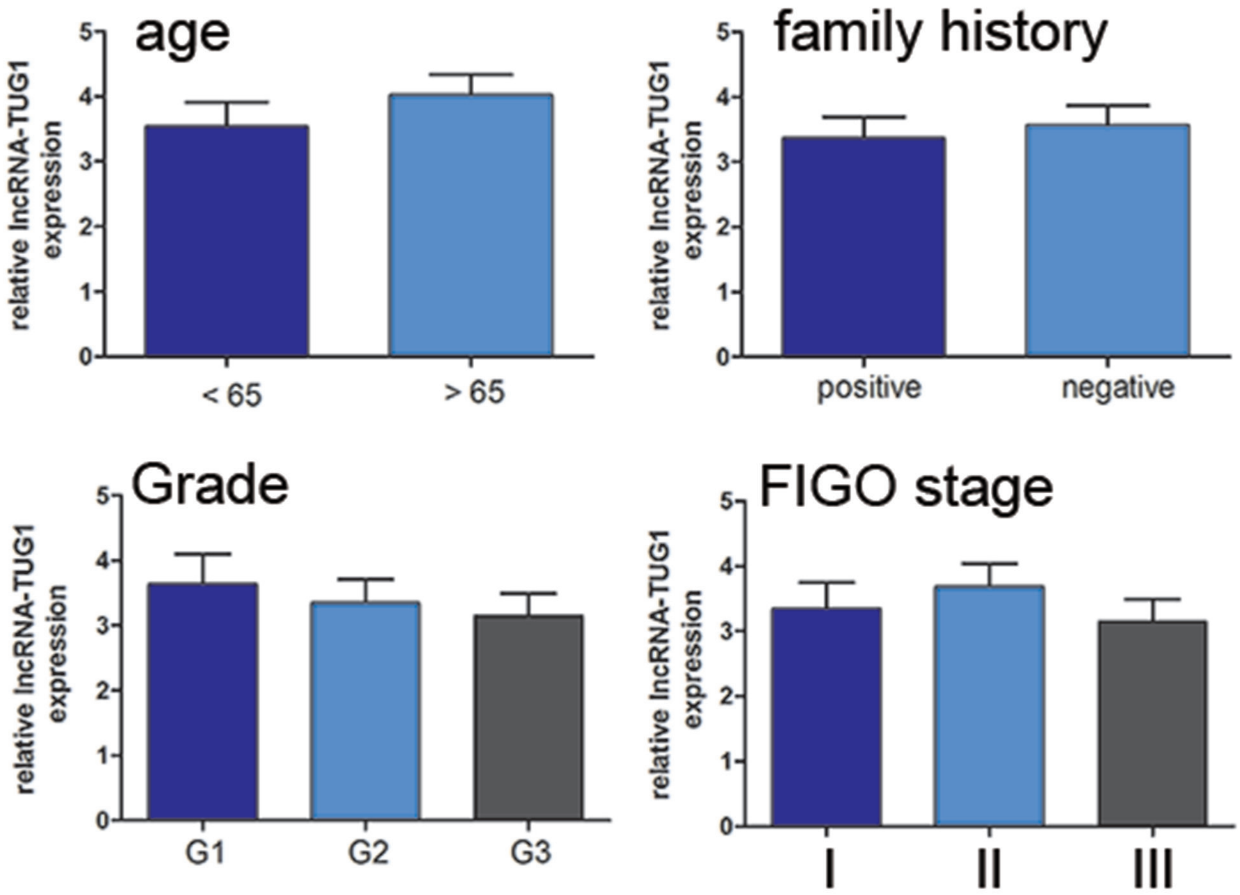

Supplementary Figure 1: lncRNA-TUG1 expression in subgroups of patients. **(A, B, C, D)** Stratification analysis of the lncRNA-TUG1 expression in subgroups of patients, there is no significance differential expression in age, family history, and grade and FIGO stage subgroups.
